# Pectin Structural Dynamics: Developmental and Evolutionary Perspectives

**DOI:** 10.3390/plants15162531

**Published:** 2026-08-21

**Authors:** Zúñiga-Sánchez Esther, Corral-Castrejón Estela, Gamboa-deBuen Alicia

**Affiliations:** 1 Facultad de Ciencias, Universidad Nacional Autónoma de México, Mexico City 04510, Mexico; luz_estelar@ciencias.unam.mx; 2 Instituto de Ecología, Universidad Nacional Autónoma de México, Mexico City 04510, Mexico; ezuniga@ecologia.unam.mx

**Keywords:** cell wall, homogalacturonan (HG), pectin dynamics, evolution, development

## Abstract

Plant cell walls play a crucial role in plant development and evolution. This structure is primarily composed of proteins and polysaccharides, including cellulose, hemicellulose, and pectins. Homogalacturonan (HG), the most abundant pectin in the primary cell wall, is synthesized by α -1,4-D-GALACTURONOSYLTRANSFERASE (GAUT) enzymes, methylesterified, and subsequently secreted into the apoplast. The dynamics of pectin methylesterification is regulated by enzymes such as PECTIN METHYLESTERASES (PMEs) and PECTIN METHYLESTERASE INHIBITORS (PMEIs). Across plant evolution and development, different cell types display distinct domains of pectin methylesterification. The binding of de-methylesterified pectins to the *Catharanthus roseus* RECEPTOR-LIKE KINASE 1-LIKE (CrRLK1L) proteins and RAPID ALKALINIZATION FACTOR (RALF) peptides is involved in pectin signaling and cell wall integrity maintenance during developmental processes. While phylogenetic studies highlight molecular innovations in pectin metabolism, functional studies remain scarce outside of angiosperms. Furthermore, the explicit role of de-methylesterified pectin in coupling cell wall structure to intracellular signaling has only been demonstrated in angiosperms. Comparative functional studies addressing key evolutionary transitions will ultimately reveal how pectin metabolism has contributed to morphological innovations across plant evolution.

## 1. Introduction

Plant cell walls play a fundamental role in regulating cell morphology and growth during plant development. Their involvement in signaling during intercellular communication and environmental interactions has also been extensively documented [1]. Plant cell walls are primarily composed of proteins interacting with a complex matrix of polysaccharides, including cellulose, hemicellulose, and pectins [2]. Pectins constitute the most complex group of these polysaccharides and comprise homogalacturonans (HGs), rhamnogalacturonan I (RG-I), and rhamnogalacturonan II (RG-II). These molecules accumulate predominantly in primary cell walls, accounting for up to 35% of polysaccharides in eudicots and non-commelinid monocots, but only 2–10% in grass primary walls [3]. Evolutionarily, pectins appeared after the divergence of Chlorophyta and Charophyta, the closest algal lineage to land plants [4].

HG, the most abundant pectin, is an α -1,4-linked homopolymer of D-galacturonic acid (D-GalA) synthesized in the Golgi apparatus by the GALACTURONOSYLTRANSFERASE (GAUT) enzyme family. Specifically, HG:α-1,4-D-GALACTURONOSYLTRANSFERASE (HG:GalAT) catalyzes the transfer of D-GalA from UDP-D-GalA to the non-reducing end of the growing HG chain. The newly polymerized HG is subsequently modified by PECTIN METHYLTRANSFERASES (PMTs), which append a methyl group at the O-6 position. As a result of this Golgi-localized transferase activity, HGs are secreted into the apoplast with high degrees of methylesterification, reaching up to 80% [5,6,7].

Once in the cell wall, the methylesterification status of HG is dynamically regulated by PECTIN METHYLESTERASES (PMEs), which catalyze the removal of the methyl group from the C-6 position of GalA, releasing methanol and protons that result in carboxil groups that are negatively charged upon protonation. PME activity determines not only the degree of methylesterification, but also the spatial pattern of de-methylesterification, which is regulated by apoplastic pH, enzyme processivity, and cation availability. The removal of the methyl group can follow either a block-wise or random pattern. A block-wise de-methylesterification pattern results in negatively charged galacturonic acid residues that can promote cell wall stiffening through calcium ion cross-linking, which forms rigid structures known as “egg boxes” [5,6,7,8,9]. Conversely, a random de-methylesterification pattern produces more fragmented HG domains and is associated with cell wall loosening. De-methylesterified HGs also serve as substrates for pectinases like POLYGALACTURONASES (PGs) and PECTIN LYASES (PLs), whose activity also promotes pectin degradation and cell wall softening. Endo-PGs cleave internal HG:α-1,4 bonds, whereas exo-PGs remove terminal GalA residues. In muro, PME and PG activities are inhibited by PECTIN METHYLESTERASE INHIBITORS (PMEIs) and POLYGALACTURONASE-INHIBITING PROTEINS (PGIPs), respectively [10]. PME–PMEI complex stability and binding affinity are sensitive to ionic strength and pH. Structural, biochemical, and functional analyses of this complex help to understand how regulation of HG methylesterification affects cell wall mechanics.

The partial degradation of HGs generates oligogalacturonides (OGs) with diverse degrees of polymerization and de-methylesterification patterns. Both de-methylesterified pectin and OGs can interact with wall-associated sensor proteins, such as RAPID ALKALINIZATION FACTOR (RALF) peptides, as well as receptor proteins including the *Catharanthus roseus* RLK1-LIKE (CrRLK1L) family, WALL-ASSOCIATED KINASES (WAKs), and LEUCINE-RICH REPEAT KINASES (LRRK) [11,12,13,14].

The involvement of HGs with varying degrees of methyl-esterification has been documented throughout the development of charophyte algae, Bryophytes, and Lycophytes [4,15,16]. For instance, egg-box formations and the contribution of pectin to cell wall integrity have been described in the alga *Penium margaritaceum* [17,18]. Furthermore, *GAUT* family genes involved in HG synthesis have been identified in charophyte algae [19,20]. The evolutionary origins of PMEs and PGs are consistent with the emergence of pectins in early charophyte cell walls [21,22]. While *PMEI* genes have been described in both Bryophytes and Lycophytes, suggesting that land plants acquired early mechanisms to fine-tune apoplastic PME activity, *PGIP* genes remain undetected in the Lycophyte *Selaginella moellendorffii* [23].

Although genome sequences for various ferns (*Azolla filiculoides*, *Salvinia cucullata*, *Ceratopteris richardii*, *Adiantum capillus-veneris*, and *Alsophila spinulosa*) are now available, no phylogenetic studies focused on ferns have been conducted for protein families related to HG synthesis and chemical modification [24,25,26,27]. Finally, while *CrRLK1K* genes are present in charophytes, *RALF* family genes appear to be absent in this group [28].

## 2. Synthesis and Methylesterification of Homogalacturonans

### 2.1. Galacturonosyltransferase (GAUT) Family

In charophyta, six genes belonging to the *GALACTURONOSYLTRANSFERASE* (*GAUT)* family have been identified [20]. The number of *GAUT* homologues increases to nine in the Bryophyte *Physcomitrium patens* and ten in the Lycophyte *Selaginella moellendorffii* [19]. In contrast, the *Arabidopsis thaliana* (Arabidopsis) genome harbors 15 members of the *GAUT* family [29] (Table 1).

Biochemical, cellular, and functional characterizations of enzymes involved in homogalacturonan (HG) synthesis have been predominantly performed in spermatophytes, particularly Arabidopsis. The HG-synthesizing activity of Arabidopsis GAUT1 was biochemically characterized using suspension cell cultures [40]. In vitro activities have also been confirmed for enzymes encoded by *GAUT4*, *GAUT7*, *GAUT10*, *GAUT11*, *GAUT13*, and *GAUT14* in Arabidopsis and other dicot species [41,42]. Consequently, GalA or total pectin content is altered in all described *gaut* mutants. Subcellular localization of GAUT proteins has been successfully mapped only in Arabidopsis, demonstrating that at least seven of these proteins reside in the Golgi apparatus. The Golgi targeting of GAUT1 is mediated by its interaction with GAUT7 [7,41]. Additionally, the scaffold protein ELMO1 promotes the assembly of a GAUT8/GAUT9/QUASIMODO2 (QUA2)/ELMO complex that regulates pectin accumulation and hypocotyl cell adhesion [43].

Most *GAUT* genes are ubiquitously expressed across plant tissues, with the exception of *GAUT11*, whose expression is restricted to seeds, where it modulates seed coat mucilage content [41,44]. Six *GAUT* genes highly expressed in stamens are critical for pollen germination; notably, a male infertility phenotype has been observed in the *gaut5/gaut6/gaut7* triple mutant [7]. Furthermore, pollen-expressed *GAUT13* and *GAUT14* exhibit redundant functions in maintaining pollen tube shape and regulating growth [45].

*GAUT* genes are also implicated in vegetative growth. For example, *GAUT10* is an auxin-responsive gene that regulates sugar-mediated root meristem maintenance; accordingly, *gaut10* seedlings develop short roots in the absence of sucrose. *GAUT8/QUA1* and *GAUT12* are essential for cell adhesion and cell wall integrity, and *gaut8/gaut12* double mutants display severe dwarfism [46,47,48,49,50]. Stomatal dynamics are also affected, showing an increase in the *gaut10/gaut11* double mutant [51] (Table S1).

### 2.2. Homogalacturonan:Methyl Transferase (HG:MT) Family

Although methylesterified HGs occur in charophyte cell walls, no information is available regarding the presence of *PMT* genes. The PMT catalyze the transfer of methyl groups from S-adenosyl-L-methionine (SAM) to the C-6 carboxyl groups of newly synthesized homogalacturonan. In Bryophytes, three and one putative *PMT* genes have been identified in *P. patens* and *Marchantia polymorpha*, respectively, whereas three have been reported in *S. moellendorffii* [23,32]. In Arabidopsis, the HG:MT family contains 29 members [35] (Table 1).

*QUASIMODO2* (*QUA2/TUMOROUS SHOOT DEVELOPMENT 2*, *TSD2/THINGS FALL APART2*, *TFA*) is the most thoroughly characterized gene within this family. PMT activity has been detected in purified Golgi fractions and crude membrane extracts from various plants [52,53,54]. In vitro biochemical methyltransferase activity was definitively demonstrated using purified QUA2 recombinant protein [55]. *QUA2* is highly expressed in most tissues, particularly in meristems and young organs, but is absent in mature pollen [55]. *tsd2/qua2* mutants exhibit increased axial meristem activity, reduced root growth, and enhanced de-etiolation [35]. Cell walls of *qua2* mutants display reduced HG content and compromised cell adhesion, while *tfa/qua2* mutants exhibit short hypocotyls and defective seed mucilage [55,56]. Furthermore, transcriptome analysis of etiolated *qua2* seedlings revealed the downregulation of cell wall-related kinases involved in signaling and integrity, such as *THESEUS1* (*THE1*) [57]. QUA3 is a Golgi-localized protein similar to QUA2 with methyltransferase homogalacturonan methyltransferase activity. QUA3 influences pectin methylesterification and regulates cell wall biosynthesis. *QUA3* RNA interference (RNAi) knockdown suspension-cultured cells have less pectin methylation, and cell wall polysaccharide composition and assembly are altered. The increase in de-methylesterified pectin is likely related to *PME* expression. The genes *At1g02810* and *At1g11580*, two of the most highly expressed *PME* genes in suspension-cultured cells, showed a decrease in expression, while the gene *At5g53370* exhibited a moderate increase in expression in *qua3i* cells. However, reduced de-methylesterification levels in the RNAi line could be related to QUA3 decrease [58]. Finally, another *PMT* member, *At2g45750*, is one of the 100 most up-regulated genes in root hairs compared to non-root hair tissues and is specifically regulated by IAA [59] (Table S2).

A distinct family of putative methyltransferases, characterized by a bacterial-like methyltransferase domain, is conserved across both monocots and dicots [60]. In Arabidopsis, this family includes COTTON GOLGI RELATED 2 (CGR2) and CGR3. A decrease in pectin methyl-esterification is observed in *cgr3* mutant cell walls, whereas *CGR3*-overexpressing plants show the opposite effect [54,60]. These genes are required for hypocotyl and petiole elongation [54,61]. Additionally, *CGR3* overexpression in tobacco enhances foliar photosynthetic CO_2_ uptake [62]. To date, direct biochemical evidence of methyltransferase activity remains unestablished for CGR2 and CGR3 proteins.

## 3. De-Methylesterification of Homogalacturonans and Its Regulation

### 3.1. Pectin Methylesterases (PMEs)

PMEs are widely distributed across plants and microorganisms, including fungi and bacteria [63]. In land plants, PMEs are classified into two groups. Type I (or PRO-PMEs) possess two domains: an N-terminal PMEI-like domain, homologous to *PMEI* genes from kiwifruit (*Actinidia deliciosa*) and Arabidopsis, and a C-terminal catalytic domain corresponding to the mature, active isoform found in the cell wall [63,64]. A serine protease processes the PRO-PME domain within the Golgi apparatus to facilitate extracellular targeting, representing a post-translational mechanism that regulates PME activity [6,10]. In contrast, Type II PMEs contain only the catalytic domain.

The charophyte *Coleochaete orbicularis* possesses 15 Type II *PME* genes encoding a single catalytic domain, whereas land plants contain both PME types [10,21,64]. The PRO-PME domain is present in 12 of the 47 *PME* genes in the Bryophyte *P. patens*, while the Arabidopsis genome encodes 23 Type II and 43 Type I PMEs [21] (Table 1). PME activity has been detected in both developing and mature cells of the unicellular charophyte *Micrasterias denticulata*, with enzymatic assays revealing multiple distinct PME isoforms [65].

PME biochemical activity has been validated using enzymes purified from various plant tissues, establishing distinct substrate specificities and pH optima [66,67,68]. In Arabidopsis, the recombinant enzymes PME2, PME3, PME31, and PPME1 have been extensively characterized. For PME2, an HG esterification degree of 55–70% is optimal. PME31 displays an optimal pH of 8.0–8.5, whereas PME3 and PME2 operate optimally between pH 7.0 and 7.5 [69,70,71,72,73]. Furthermore, PME2 exhibits a processive (continuous) mode of action at pH 8.0, but switches to a non-processive (random) cleavage pattern at pH 5.0 [73].

Expression profiling has revealed distinct tissue- and stage-specific patterns for *PME* genes in Arabidopsis [74,75], a phenomenon conserved across diverse plant species [76,77]. In Arabidopsis, 16 ubiquitously expressed *PMEs* participate in stem, hypocotyl, and root development. For instance, *pme35* mutants exhibit impaired mechanical stem strength, while *pme3*, *pme17*, *pme44*, and *pme2* mutants display altered hypocotyl and root elongation [71,73,78,79]. In *pme3* mutants, a reduction in root hairs and an increase in adventitious root formation have been observed relative to wild-type plants [70,80]. PME3 also regulates pavement cell morphogenesis and cell wall integrity; *pme3* cotyledons show interdigitation defects, and the lobes and necks are completely absent in *pme2/pme3* double mutants [81,82].

HG de-methylestesterification also modulates guard cell wall function [83]. The *pme2/pme3* double mutant shows a significant increase in mature stomata numbers compared to the wild type [82]. Conversely, enrichment of methyl-esterified pectin in the guard cell walls of *pme6-1* mutants impairs stomatal opening and closure [84]. PME12, PME34, and PME53 are also implicated in stomatal regulation under heat stress [85,86,87].

In the shoot apical meristem (SAM), newly formed cell walls in dividing cells are enriched with de-methylesterified pectin, whereas methylesterified pectin predominates in mature cells. *PME5* is expressed in mitotic cells and plays a critical role in establishing and maintaining these two distinct pectin domains, which in turn are essential for defining the cell division plane [88].

The specific expression of certain PME isoforms in reproductive tissues is well documented in angiosperms [74,89]. Fourteen *PME* genes in Arabidopsis and eleven in rice exhibit high or pollen-specific expression, driving pollen development, germination, and tube elongation [76,90,91]. For example, PME48 remodels HG within the pollen intine cell wall [91]. In *qrt1* mutants, pollen grains fail to separate and remain as tetrads, indicating that QUARTET1 is required for pollen grain separation [92]. In *pme5*/*vgd1* and *pme4*/*vgdh2* mutants, altered pollen tube elongation results in shortened siliques and reduced seed sets [93]. Similarly, *ppme1* mutants exhibit defective pollen tube elongation [94]. In maize, two pollen-expressed *PMEs*, *ZmGa1P* and *ZmPME10-1*, modulate the cross-incompatibility response [95].

PME-mediated pectin remodeling is equally vital during seed-coat mucilage development. *pme58* mutants show reduced seed mucilage [96] (Table S3).

### 3.2. Pectin Methylesterase Inhibitors (PMEIs)

Phylogenetic studies of the PME and PMEI families suggest that PMEI domains evolved from the PRO-PME domain. Consequently, no PMEI genes have been detected in Charophytes. A single *PMEI* gene has been described in *P. patens* and *S. moellendorffii*, whereas large, multigenic *PMEI* families have expanded in angiosperms, with 71 members identified in Arabidopsis [21] (Table 1).

PMEI proteins were initially characterized as competitive inhibitors of PME activity in kiwi (*Actinidia chinensis*) [97,98]. Subsequently, the inhibitory profiles of recombinant PMEI1, PMEI2, PMEI4, PMEI7, PMEI18, and PMEI9 have been characterized in vitro. This inhibitory effect can be pH-dependent or pH-independent [71,99,100,101,102]. For example, PMEI4 and PMEI7 inhibit AtPME3 activity at pH 5.0 but leave it unaltered at pH 7.5 [71,101]. Similarly, the inhibition of PME2 and PME3 by PMEI3 is pH-dependent [102]. Conversely, PMEI9-mediated inhibition of PME2 and PME3 operates independently of pH [73,101]. PMEI1 and PMEI2 also interact directly with PME3 [103], while PMEI18 specifically inhibits PME31 activity [104].

*PMEI* gene expression patterns vary across the gene family; while some family members are broadly expressed throughout development, many exhibit strict tissue specificity [105,106,107]. In Arabidopsis, *PMEI4*, *PMEI8*, and *PMEI9* are expressed in vegetative tissues. *PMEI4* is highly upregulated in the epidermis during dark-induced hypocotyl elongation [108], whereas *PMEI8* and *PMEI9* modulate root growth under cellulose-deficient conditions [109]. Furthermore, increased pectin de-methylesterification in the guard cells of *pmei8* mutants impairs stomatal responsiveness to environmental cues [104].

Finally, seed-coat mucilage de-methylesterification and release are controlled by PMEI6, PMEI13, and PMEI14. *pmei6* mutants show delayed mucilage release upon imbibition alongside elevated total seed PME activity [110]. Although *pmei13* and *pmei14* seeds display decreased mucilage de-methylesterification and increased PME activity, their overall mucilage release remains unaffected (Table S4).

## 4. Functional Analysis of Pectin Methylesterification Status

Experimental alterations of the methyl-esterification status of pectins, utilizing overexpression lines of PECTIN METHYLESTERASES (OEPMEs) and/or PME INHIBITORS (OEPMEIs), have elucidated the functions of these protein families in seedling emergence, flowering time, and pollen tube elongation. Epigallocatechin gallate (EGCG) is an antioxidant molecule that also inhibits PME. These approaches have helped to determine the role of de-methylesterified pectins in cell signaling processes.

Seedling emergence is mediated by the Arabidopsis hypocotyl. Ectopic expression of *PMEI5* markedly increases homogalacturonan (HG) methyl-esterification levels in epidermal cell walls relative to wild-type (WT) cells, thereby attenuating the characteristic asymmetry in HG methyl-esterification between outer and inner epidermal cell faces. Consequently, *PMEI5*-overexpressing (*OE-PMEI5*) seedlings fail to emerge when buried in soil [111]. In Arabidopsis stems, the presence of phloem sieve elements is increased in *OEPME5* plants but decreased in *OEPMEI1* plants. As a result, the transport efficiency of the florigen FLOWERING LOCUS T increases in *OEPME5* plants, promoting earlier bolting [112]. Regarding reproductive development in Arabidopsis, the addition of recombinant PMEI9 to culture media promotes pollen tube growth [101]. Moreover, in epidermal root cells, EGCG inhibition of RALF-induced extracellular pH alkalinization has been detected, indicating that PME activity is directly involved in signaling mechanisms [113].

Domain of Unknown Function 642 (DUF642) proteins also participate in regulating methylesterification across various developmental processes [114]. The DUF642 family of cell wall proteins is specific to spermatophytes [115]. Arabidopsis *TEEBE* (*TEB*) gene is expressed in seedlings and carpels. The hypocotyl cell walls of *teb* seedlings exhibit higher levels of methylesterified pectins than the WT; consequently, both their hypocotyl cells and the hypocotyls themselves are longer than those of the WT [116]. In rice, the DUF642 protein Diurnal Flower Opening Time 1 (OsDFOT1) is involved in regulating the methylesterified pectin status of lodicule cells to control cell swelling. Accordingly, *Osdfot1* mutants exhibit earlier diurnal flower opening. Furthermore, physical interactions between OsDFOT proteins and multiple PMEs have been demonstrated [117]. Similarly, the Arabidopsis DUF642 protein BIIDXI, which is involved in seed production, interacts directly with PME3 [118].

## 5. Demethylesterified Pectin-Binding Peptides and Proteins

### 5.1. Catharanthus Roseus Receptor-like Kinase 1-like (CrRLK1L) Proteins

CrRLK1Ls are modular proteins featuring an extracellular domain with a two-tandem malectin domain (MD), a transmembrane domain, and a cytosolic kinase domain. The MD binds distinct molecules or co-receptors, while the kinase domain activates downstream proteins involved in mediating specific responses [30,119]. For example, the interaction of rapid alkalinization factors (RALFs) with CrRLK1Ls induces rapid physiological responses, such as changes in cytosolic calcium levels and extracellular alkalinization [120].

Evolutionarily, seven *CrRLK1L* genes have been described in charophytes. In land plants, early-diverging Bryophytes present one to six genes, whereas Lycophytes harbor two. Among vascular plants, five to ten *CrRLK1Ls* have been identified in fern genomes, two to nine in gymnosperms, and up to 42 in angiosperms [28,30,31]. Functional studies of MpFERONIA (MpFER), the sole CrRLK1L described in *Marchantia polymorpha*, demonstrated that this receptor controls cell wall integrity (CWI) during rhizoid tip growth [121]. Indeed, *mpfer* mutants display short rhizoids, with overall plant growth being severely inhibited by abscisic acid (ABA) treatment [122].

The roles of CrRLK1Ls in vegetative development and plant reproduction have been extensively documented across various vascular plants [30]. In Arabidopsis, seventeen CrRLK1Ls have been identified. FERONIA (FER), the first and most comprehensively studied CrRLK1L, is expressed in all plant organs except anthers and pollen [123]. Consequently, *fer* mutants present a dwarfed phenotype characterized by short hypocotyls, cotyledons, and roots. A decrease in seed production, driven by defects in female signaling, has also been reported for *fer* mutants [119].

While FER, THESEUS (THE), and ERULUS (ERU) are required for root hair development and elongation [124,125], ANXUR (ANX) and BUDDHA PAPER SEAL (BUPS) participate in maintaining cell wall integrity during pollen tube elongation [126].

The binding of RALF peptides to CrRLK1L proteins and alternative targets has been widely characterized [127]. The RALF1–FER complex inhibits root elongation by promoting apoplastic alkalinization, whereas RALF22–FER regulates root hair development [120,125]. Additionally, the interaction of RALF34 with THE is involved in lateral root initiation [128].

**Figure 1:**
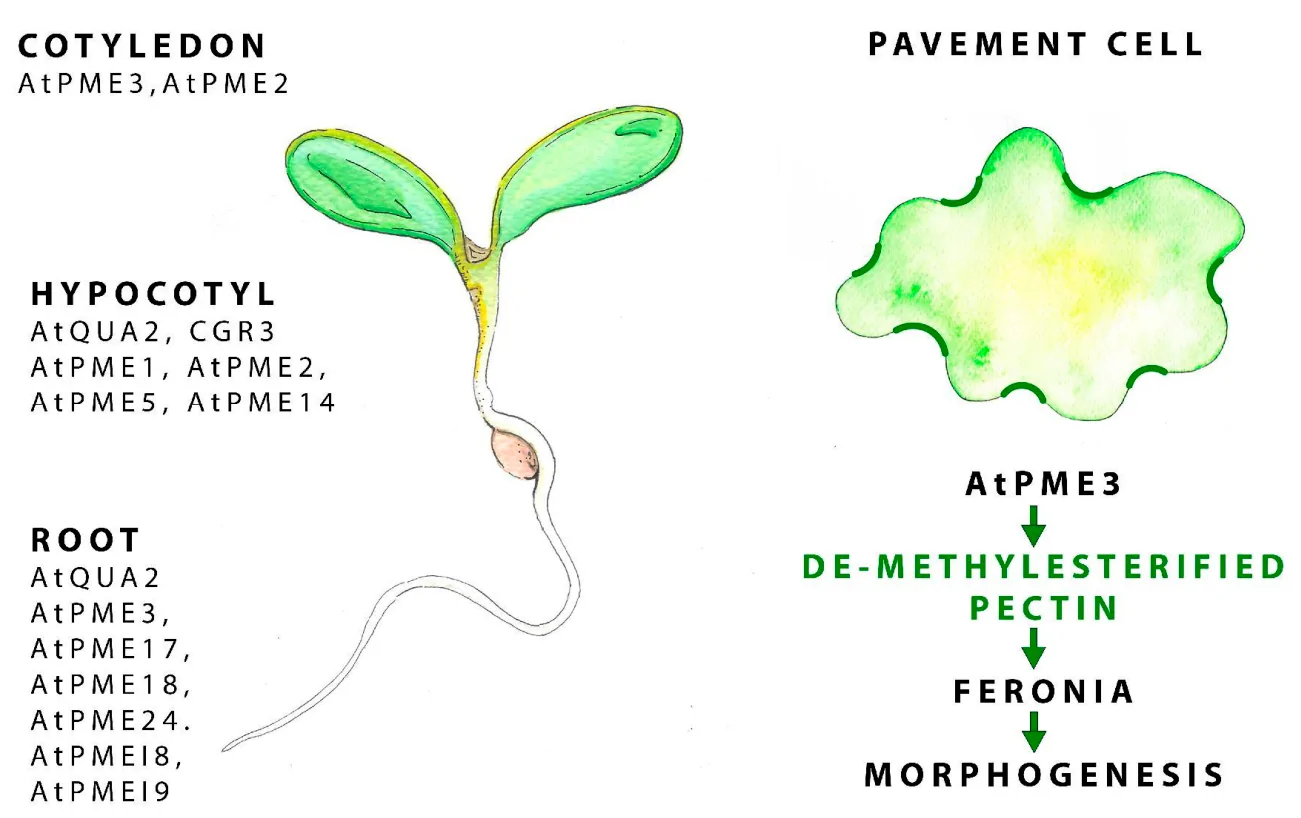
Overview of the roles of pectin dynamics-related enzymes in *Arabidopsis thaliana* seedling development. METHYL TRANSFERASE (HG:MT), PECTIN METHYLESTERASES (PMEs), and PME INHIBITORS (PMEIs) participate in hypocotyl elongation and root growth. Functional studies have demonstrated that PME2 and PME3 are involved in cotyledon development. Specifically, AtPME3 contributes to the establishment of two cell-wall domains with distinct pectin properties to promote pavement cell (PC) morphogenesis. De-methylesterified pectin activates the FERONIA signaling pathway to initiate lobe formation. In pavement cells, highly de-methylesterified pectin within the necks is represented in dark green. Arrows indicate the contribution of the PME3 enzyme to the formation of a de-methylesterified cell-wall microdomain and the binding of de-methylesterified pectin to FERONIA to activate PC morphogenesis.

The direct interaction between the MD of FER and de-methylesterified HGs has been demonstrated during pavement cell (PC) morphogenesis. The binding of de-methylesterified pectins to FER activates signaling cascades that promote PC lobe formation; consistently, the cell walls at PC necks are highly enriched in de-methylesterified pectins. The pavement cells (PCs) of the *pme3* mutant display interdigitation defects. Pectin is highly de-methylesterified in the neck cell wall [81] (Figure 1).

FER and ERU also participate in modulating the activity of pectin-modifying enzymes in ovules, hypocotyl epidermal cells, and root hair cells [124,129,130]. In *fer* mutants, an increase in PME activity drives a decrease in methylesterified pectins alongside an increase in hypocotyl epidermal cell volume [130]. In ovules, FER-mediated accumulation of de-methylesterified pectin at the filiform apparatus acts as a mechanism to prevent the entry of supernumerary pollen tubes into the female gametophyte [129]. Conversely, the apical localization of ERU correlates with the deposition of highly methylesterified pectins in root hairs. The short root hair phenotype of *eru* mutants is determined by elevated PME activity at the root tip [124].

### 5.2. Rapid Alkalinization Factor (RALF) Peptides

RALFs are small, basic peptides characterized by eight polar, positively charged amino acid residues [131]. Phylogenetic studies indicate that the RALF family is absent in charophytes. However, Bryophytes and Lycophytes possess three and four *RALF* genes, respectively, suggesting that this family emerged during the transition to land plants. The copy number of *RALF* genes expands significantly in vascular plants, with four found in ferns, seven to 13 in gymnosperms, and up to 37 in angiosperms [33,34,132]. Functional characterization of the three *P. patens* genes revealed that *PpRALF1* and *PpRALF2* promote tip growth and cell elongation in the protonema [34].

In Arabidopsis, *RALF* genes exhibit widespread expression profiles across both vegetative and reproductive tissues, though a substantial number are exclusively expressed in the inflorescence and flowers [33,133]. At least 15 genes are expressed in pollen, where they regulate pollen tube elongation and pollen-pistil interactions [134,135]. RALF peptides are also critical for root growth and development, including root hair formation [136,137].

**Figure 2:**
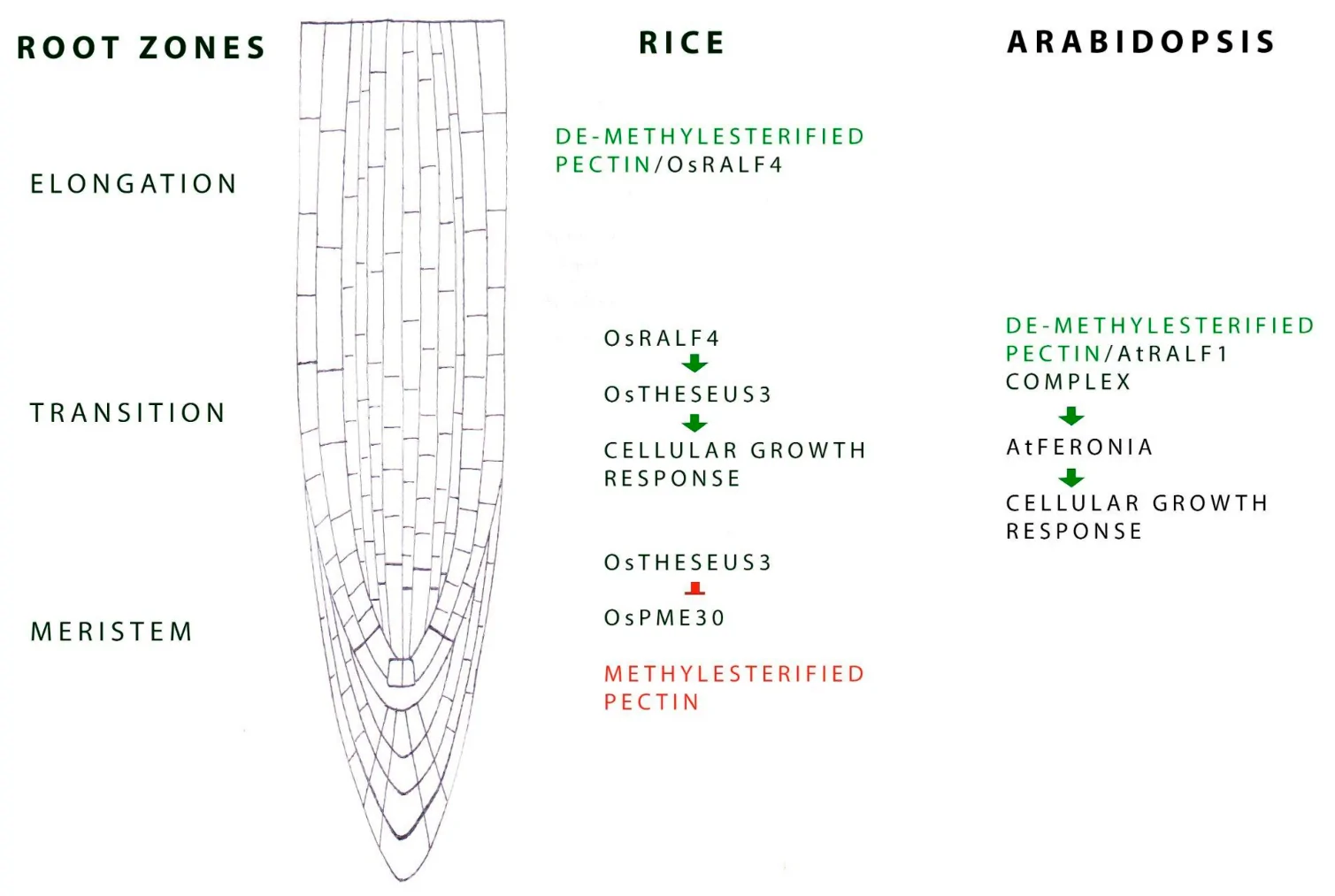
Molecular interactions driving cell growth and cell wall structural remodeling in *Oryza sativa* (Rice) and *Arabidopsis thaliana* (Arabidopsis) root elongation. Rice root: Highly methylesterified pectins are abundant in the meristematic zone, where PME30 activity is inhibited by the OsTHESEUS3 signaling pathway. In the transition zone, the interaction of OsRALF4 with OsTHESEUS3 promotes PME30 activity and meristematic cell expansion, coinciding with the detection of de-methylesterified pectin. A de-methylesterified pectin/OsRALF4 structural complex is formed within the cell wall of the elongation zone. Arabidopsis root: The interaction of de-methylesterified pectin and AtRALF1 with FERONIA promotes cellular growth to establish the transition zone. Green arrows indicate the positive contribution of interactions between RALF and CrRLK1L proteins in inducing cellular responses, whereas the red symbol represents the inhibition of PME activity.

The binding of RALF peptides to de-methylesterified pectins is charge-dependent, effectively bridging molecular signaling and structural processes within the cell wall during plant growth [138]. The binding of OsRALF4 to de-methylesterified HG has been mapped to the elongation zone of the primary root in rice. At the onset of meristematic cell expansion, de-methylesterified HG and OsRALF4 are also detected [139] (Figure 2). In Arabidopsis, the RALF1 and RALF22 peptides bind to de-methylesterified HG in late meristematic epidermal cells and root hair cells of the primary root, respectively [113,125]. Within root cells, the interaction of RALF with de-methylesterified pectin is required for FER engagement and the subsequent activation of complex intracellular signaling cascades that govern root growth. Thus, a scaffold function for de-methylesterified pectins has been proposed in these domains [113] (Figure 2).

**Figure 3:**
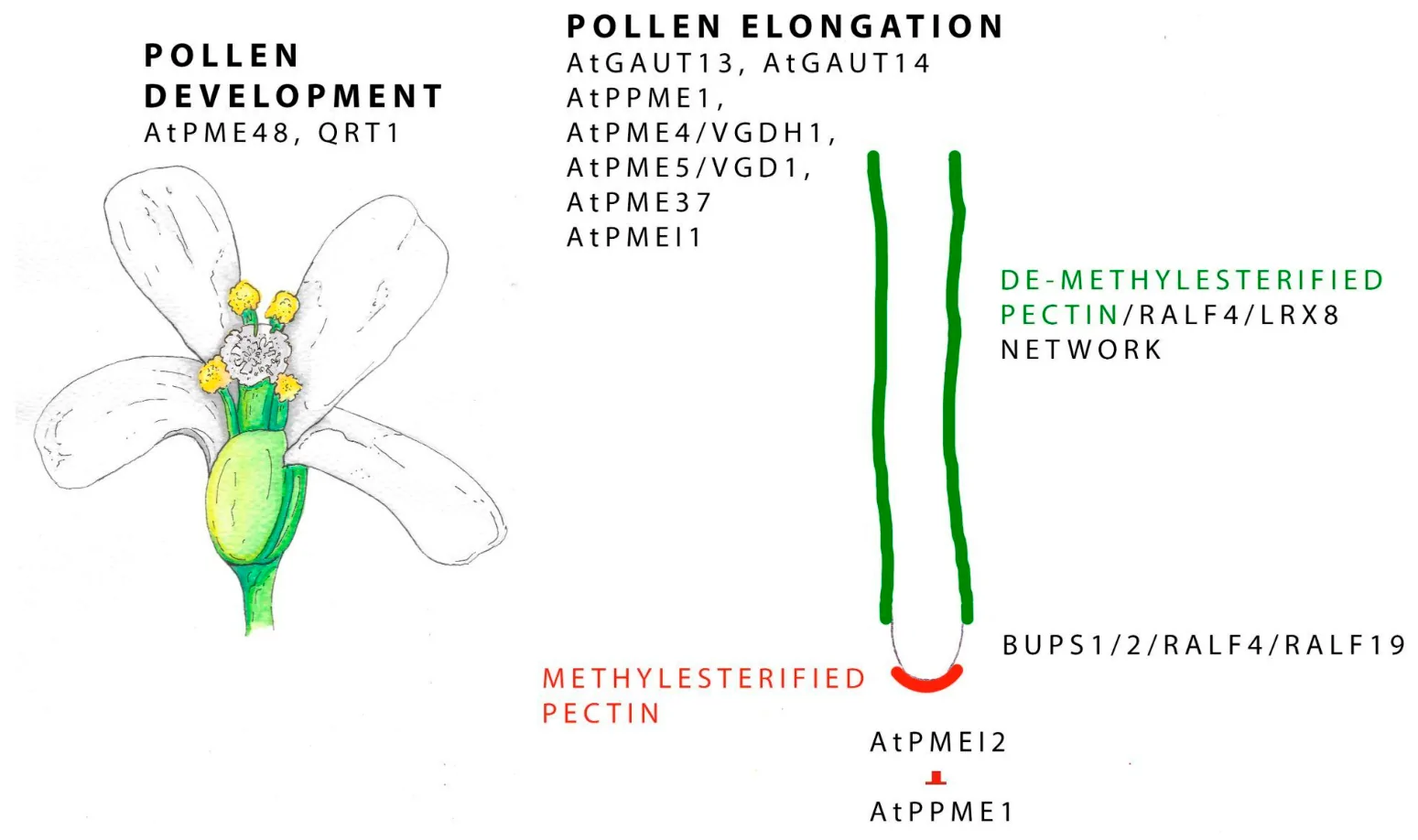
Overview of the roles of pectin dynamics-related enzymes in pollen development and elongation in *Arabidopsis thaliana*. PECTIN METHYLESTERASES (PMEs) are involved in pollen development, whereas GALACTURONOSYLTRANSFERASES (GAUTs), PMEs, and PME INHIBITORS (PMEIs) participate in pollen tube elongation. Methylesterified pectins, AtPMEI2, and AtPPME1 are secreted into the apoplast at the apex of the elongating tube. The methylesterified pectin domain at the tip is maintained through the inhibition of AtPPME1 activity by AtPMEI2. In the transition zone, endocytosis of PMEIs leads to an increase in PME activity. Furthermore, the interaction of RALF peptides with BUDDHA’S PAPER SEAL (BUPS) receptors promotes the cellular growth response. Concurrently, a cross-linked network composed of LEUCINE-RICH REPEAT EXTENSIN 8 (LRX8), RALF4, and de-methylesterified pectin is formed along the pollen tube shank, which is essential for maintaining cell wall integrity during rapid tube growth. The methylesterified pectin domain at the pollen tube tip is shown in red, and de-methylesterified pectin in the shank is shown in dark green. The red symbol denotes the inhibition of AtPPME1 activity by AtPMEI2.

During cell tip growth, the interaction of de-methylesterified pectin with LEUCINE-RICH REPEAT EXTENSIN (LRX)/RALF22 complexes provides compact structural support. The LRX1–RALF22–HG complex assembles into periodic circumferential rings that maintain cell wall integrity during root hair elongation [125]. Similarly, the LRX8–RALF4–pectin complex regulates pollen tube elongation, where the interaction of LRX8–RALF4 with de-methylesterified pectin generates a reticulated network in the cell wall of the pollen tube shank [140] (Figure 3).

## 6. Conclusions and Perspectives

In recent years, the role of pectin dynamics in plant morphogenesis—specifically in mediating the crosstalk between cell wall structure and signaling pathways—has been well established across various developmental processes in angiosperms, primarily in *Arabidopsis thaliana*. The interaction of de-methylesterified pectins with proteins and peptides involved in signaling and cell-wall integrity (CWI) maintenance highlights a dual functional role for these polysaccharides.

Land plant evolution is characterized by key morphological and molecular innovations. Mechanisms for CWI maintenance, apical growth, branching, and rooting all preceded the origin of terrestrial plants, with early land plants displaying an expansion of CWI monitoring systems dedicated to perceiving and adapting to cell wall modifications during development. Although the relevance of pectin in cell expansion and CWI maintenance has been documented in charophytes, which possess pectin biosynthetic and *PECTIN METHYLESTERASE* (*PME*) genes but lack *PECTIN METHYLESTEREASE INHIBITORS* (*PMEIs*), its precise role in early land plants remains poorly understood.

In tip-growing cells such as protonemata and rhizoids, pectin localization suggests that pectin dynamics contribute to apical growth; however, distinct domains enriched in methylesterified versus de-methylesterified pectins remain ill-defined. Similarly, gymnosperm pollen tubes, particularly in conifers, lack the spatial segregation of methyl-esterification status characteristic of angiosperm pollen tubes. This structural difference correlates with growth kinetics: protonemata and conifer pollen tubes exhibit slow tip growth, whereas angiosperm pollen tubes—and those of select gymnosperm lineages—display rapid elongation [141]. Crucially, the functional roles of pectin-remodeling enzymes and de-methylesterified pectin interactors during elongation in *Marchantia polymorpha* protonemata and gymnosperm pollen tubes remain uncharacterized. Investigating these enzymatic networks across diverse lineages will be pivotal to understanding the evolutionary trajectories of tip-growing cells.

The emergence of PMEIs and RAPID ALKALINIZATION FACTORS (RALFs) in land plants represents a key evolutionary innovation for the in muro regulation of methyl-esterification and CWI maintenance, respectively. Future functional analyses will be essential to elucidate how interactions among de-methylesterified pectins, RALFs, and *Catharanthus roseus* RLK1-LIKE (CrRLK1L) proteins evolved alongside the morphological and physiological diversification of land plants.

In early land plants, stomata are present in several moss genera and represent a key morphological innovation. Stomata have been secondarily lost in liverworts [142]. In Arabidopsis, guard cell walls contain two distinct pectin domains established through heterogeneous deposition and in muro modification, which are essential for stomatal morphogenesis and dynamics. However, the precise role of pectin in stomatal signaling and CWI maintenance remains largely unexplored.

Similarly, despite the paramount importance of vascular tissue for plant diversification, the contribution of pectin dynamics to vascular development and function remains understudied, even in model systems like Arabidopsis. A significant knowledge gap persists regarding pectin metabolism genes and their developmental roles in early vascular lineages, such as lycophytes and pteridophytes. Functional and biochemical studies across early vascular plants and spermatophytes are urgently needed to provide direct evidence of how pectin dynamics drive vascular evolution.

Overall, while pectin dynamics are clearly implicated in plant development across lineages, their explicit role in coupling cell wall structure to intracellular signaling has only been demonstrated in angiosperms. A major outstanding question is when this signaling capacity first arose as a molecular innovation. Although foundational signaling components (such as CrRLK1L proteins) are present in early lineages like charophytes, functional validation is lacking, especially given that RALF genes appear absent in charophyte genomes. For example, while lobe formation in the unicellular charophyte *Micrasterias denticulata* mirrors the pectin distribution patterns seen in angiosperm pavement cells (high methyl-esterification at lobes and de-methylesterified pectins at necks), the involvement of signaling components remains unknown. Comparative functional studies addressing key evolutionary transitions will ultimately reveal how pectin metabolism has contributed to morphological innovations across plant evolution.

## Figures and Tables

**Table 1:** Occurrence of pectin-related proteins across plant lineages.

Group	Representative Species	GAUT	PMT	PME	ProPME-PME	PMEI	CrRLK1L	RALF
**Charophyta** [20,21,28,29,30,31]	*Nitella mirabilis*	6	*	*	*	0	1	0
	*Coleochaete orbicularis*	d	*	15	0	0	0	0
	*Closterium peracerosum-strigosum-* *littorale* Complex	d	*	*	*	*	1	0
	*Chara brownie*	*	*	d	*	*	7	0
**Bryophyta** [19,21,23,28,30,32,33,34]	*Physcomitrium patens*	9	3	35	12	1	6	3
	*Marchantia polymorpha*	5	1	14	*	0	1	3
**Lycophyta** [19,21,23,28,30]	*Selaginella moellendorffii*	10	3	18	5	1	2	4
**Pteridophyta** [30]	*Azolla filiculoides*	d	*	*	*	*	10	4
	*Salvinia cucullata*	d	d	d	*	d	5	4
**Spermatophyta**								
**Gymnosperm** [28,30]	*Picea abies*	d	d	d	*	d	9	13
	*Pinus taeda*	*	*	*	*	*	2	7
**Angiosperm**								
**Dicots** [19,21,23,28,29,30,33,35]	*Amborella trichopoda*	d	d	15	15	17	9	9
	*Arabidopsis thaliana*	15	29	23	43	71	17	37
	*Populus trichocarpa*	23	4	24	52	54	42	20
	*Vitis vinifera*	14	5	21	20	8	17	4
	*Glycine max*	79	12	127			38	23
**Monocots** [21,33,36,37,38,39]	*Oryza sativa*	39	20	23	18	35	36	14
	*Sorghum bicolor*	36	3	23	19	55	28	16
	*Brachypodium distachyon*	36	2	3	*	2	16	10

Not present in the genome of this species (0). No data available in this species (*). Detected in the genome but the information has not been used for phylogenetic studies (d).

## Data Availability

No new data were created or analyzed in this study. Data sharing is not applicable to this article.

## Supplementary Materials

The following supporting information can be downloaded at: https://www.mdpi.com/article/10.3390/plants15162531/s1. Table S1: *Arabidopsis thaliana* GALACTURONOSYLTRANSFERASE (GAUT) family: Biochemical, cellular and physiological data; Table S2: *Arabidopsis thaliana* PECTIN METHYL TRANSFERASE (PMT) family: Biochemical, cellular and physiological data; Table S3: *Arabidopsis thaliana* PECTIN METHYL ESTERASE (PME) family: Biochemical, cellular and physiological data; Table S4: *Arabidopsis thaliana* PECTIN METHYL ESTERASE INHIBITOR (PMEI) family: Biochemical, cellular and physiological data.
